# Changes in smoking due to COVID-19 pandemic among persons of migrant origin compared with the general population: a population-based study

**DOI:** 10.1177/14034948231199792

**Published:** 2023-09-19

**Authors:** Otto Ruokolainen, Eero Lilja, Hanna Ollila, Anu E. Castaneda, Päivikki Koponen, Natalia Skogberg

**Affiliations:** Finnish Institute for Health and Welfare, Department of Public Health and Welfare, Helsinki, Finland

**Keywords:** Smoking, tobacco use, COVID-19, migrant population, population-based, risk behaviour

## Abstract

**Aims:**

Prior studies have implied that smokers may have changed their smoking behaviour during the COVID-19 pandemic. However, little is known about changes in smoking behaviour and correlates of change due to the pandemic among persons of migrant origin compared with the general population.

**Methods:**

Population-based cross-sectional studies with comparable study protocols and measures, one focusing on persons of migrant origin living in Finland (*n* = 3587, response rate 60%) and the other on the general Finnish population (*n* = 3444, response rate 56%), were utilised. The outcome measure was self-reported change in smoking behaviour due to COVID-19 among current smokers. Explanatory factors included sociodemographic-, health-, and COVID-19-related factors. Multinomial logistic regression was used in the analyses.

**Results:**

Most of the current smokers reported no change in their smoking behaviour. In the adjusted model, younger age was positively associated with increased smoking, while region of origin (Russia, Africa, Asia, and Latin America) and worrying about getting infected with COVID-19 were associated with decreased smoking among persons of migrant origin. In the general population, younger age, female sex, being other than employed/student, increased loneliness, and decreased contact with close ones were associated with increased smoking, while reduced working capacity and worries that someone close to the respondent will be infected with COVID-19 were associated with decreased smoking.

**Conclusions:**

**The findings of this study contribute to better identification of at-risk populations in future crises situations. This will allow for more efficient targeting and tailoring of health promotion services, including smoking cessation.**

## Introduction

Smoking is a leading preventable cause of premature mortality and morbidity in the world [1]. There are differences in smoking across population groups, including among persons of migrant origin compared with general populations [2]. Higher prevalence of smoking has been reported among migrant origin men than women and there is heterogeneity by country of origin and location of residence [2–4].

Finland has comparatively low rates of persons of migrant origin, yet the proportion of migrant origin persons has rapidly increased over the past decades [5]. Compared with the general population, smoking rates among migrant origin men are predominantly higher, while smoking among migrant origin women is less common [4].

The COVID-19 pandemic, which started in late 2019, disrupted everyday lives due to reductions in human mobility and social contact, and an increase in time spent at home due to lockdowns. The impact of COVID-19 was more substantial for persons of migrant origin than for the general population [6]. Smoking has been associated with increased COVID-19 severity [7] and increased mortality [8,9] among COVID-19 patients, although some studies indicate smoking to be a protective factor for COVID-19-infection [10]. In addition to the immediate impact on health, the long-term health effects of COVID-19 should also be addressed. For example, anxieties related to COVID-19 may affect smoking behaviour [11]. Therefore, investigation of the changes in smoking behaviour and its correlates as a result of COVID-19 are pivotal.

COVID-19 lockdowns have been associated with both increased smoking cessation activity and increased tobacco consumption [12,13]. For instance, according to a systematic review and meta-analysis, smoking prevalence decreased during the pandemic, while half of smokers did not change their smoking behaviour [14]. However, almost all of the studies included in the review were at high risk of bias, for example due to the use of non-representative samples [14]. Similarly, a Finnish study, also with limited external validity due to its low response rate, found that most smokers did not change their smoking behaviour during COVID-19 and these changes differed little during the different time periods of COVID-19 [15]. Another study found that COVID-19 status may not be associated with reducing smoking and attempting to quit [16]; in other research, a minority of recent quit attempts were found to be triggered by COVID-19 [17]. Psychological distress has been identified as one risk factor for increased smoking during the pandemic [13,18]. Among Japanese tobacco users, fear of COVID-19 was longitudinally associated with a smaller likelihood of quitting smoking [19].

In summary, some studies have examined changes in smoking behaviour during COVID-19, mostly with non-representative samples. Often, it is unclear whether the changes that have occurred were due to COVID-19. Furthermore, factors associated with changes in smoking behaviour are understudied, particularly among persons of migrant origin. Even though pivotal from a health equity perspective, to the best of our knowledge, no prior studies have examined these changes among persons of migrant origin, nor compared the changes with the general population using population-based samples. The aim of this study was to fill this gap. Our research questions were as follows:

1. Are there changes in smoking behaviour due to the COVID-19 pandemic among persons of migrant origin living in Finland and among the general population?
2. Are the changes in smoking behaviour due to the COVID-19 pandemic similar among persons of migrant origin living in Finland and among the general population?
3. Which background factors (sociodemographic, health-related, COVID-19-related) are associated with changes in smoking behaviour due to the COVID-19 pandemic among persons of migrant origin living in Finland and among the general population?

## Methods

### Study design

Two population-based samples were utilised. Detailed descriptions of the samples are provided elsewhere [20,21]. The Survey on the Impact of the Coronavirus on the Wellbeing of the Foreign Born Population in Finland (MigCOVID) was conducted between October 2020 and February 2021 by the Finnish Institute for Health and Welfare (THL). The MigCOVID survey is a follow-up study to the Survey on Wellbeing among Foreign Born Population (FinMonik). The original stratified random sample of the FinMonik survey was drawn in 2018 from the population register and constituted 12,877 persons born outside of Finland who were living permanently in Finland. FinMonik survey participants who were still living in Finland in 2020, and had not declined from further contact during the baseline survey, were invited to participate in the MigCOVID survey (*N* = 5269). A supplementary sample of persons born in Somalia (*n* = 982) was drawn from the population register and included in the migrant sample. Of those invited to participate, the response rate was 60% (*n* = 3668). Questionnaires in the MigCOVID survey were available in 18 languages and telephone interviews were conducted by trained multilingual research assistants.

The reference group constituted a subsample of participants of the FinHealth 2017 follow-up Study (hereafter: FinHealth) belonging to the corresponding age group of the MigCOVID survey (*n* = 3444, participation rate 56%). FinHealth was also conducted by THL within a similar time frame, following a similar study protocol to the MigCOVID survey.

For comparability, 21–66-year-olds from both surveys were included in the current study. Thus, our final analytic sample consisted of 7031 respondents (MigCOVID *N* = 3587, FinHealth *N* = 3444), of which 1269 reported current smoking at the time of the surveys (MigCOVID *n* = 649; FinHealth *n* = 620). Both studies received ethical approval (MigCOVID: the Ethical Committee of the THL; FinHealth: the Ethics Committee, Hospital District of Helsinki and Uusimaa). All participants provided informed consent.

### Measures

The majority of questions in the two studies were formulated identically. Information on marital status and Finnish/Swedish language skills were unavailable for the general population (in addition to the country of origin). The information on sex, age, country of birth, and underaged children in household were register-based, all other variables were based on survey responses. The study instruments are presented as Appendix A in the Supplemental material.

### Outcome

Smoking status was classified as daily smoking, occasional smoking, former smoking, and never smoking. To maximise the power of statistical tests, daily and occasional smoking were merged to describe current smoking (yes/no). Changes in smoking behaviour were assessed as follows: Has COVID-19 or its restrictive measures affected your everyday life? (among the general population: ‘. . . compared to the time before the epidemic?’; response options: no effect/yes, decreased/yes, increased/does not apply). The variable was recoded into three groups among current smokers (decrease/no change/increase) (missing information: n = 31/0.9% among persons of migrant origin and n = 40/1.1% among the general population).

### Explanatory variables

Classification of the explanatory variables, grouped into sociodemographic-, health-, and COVID-19-related, and their descriptions are presented in Table I.

**Table I.** Distribution of the study characteristics among persons of migrant origin living in Finland and the general population.

	Persons of migrant origin, % (*N* = 3587)	*n*	General population, % (*N* = 3444)	*n*
Sociodemographic factors				
Region/country of origin				
Russia or the former Soviet Union	22.0 (19.8–24.3)	1109	N/A	
Europe, North America, Oceania	32.7 (29.8–35.8)	1005	N/A	
Middle East and North Africa	15.3 (13.2–17.7)	434	N/A	
Africa (excl. North Africa), Asia and Latin America	30.0 (27.2–32.9)	1039	N/A	
Age (years)				
21–34	34.8 (31.9–37.8)	1085	29.4 (26.5–32.4)	524
35–44	30.4 (27.6–33.3)	1111	23.7 (22.0–25.5)	760
45–54	20.4 (18.2–22.8)	765	21.0 (19.5–22.6)	842
55–66	14.4 (12.5–16.6)	626	25.9 (24.3–27.5)	1318
Sex				
Male	51.8 (48.7–54.8)	1603	50.5 (48.1–53.0)	1586
Female	48.2 (45.2–51.3)	1984	49.5 (47.0–51.9)	1858
Education				
Comprehensive school or lower (Low)	13.2 (11.2–15.4)	377	6.5 (5.7–7.4)	270
Upper secondary education (Middle)	46.1 (43.0–49.2)	1274	49.8 (47.4–52.1)	1750
High education (High)	40.8 (37.8–43.8)	1882	43.8 (41.4–46.2)	1400
Employment status				
Employed full-time or part-time	65.6 (62.7–68.5)	2291	70.8 (68.4–73.1)	2391
Student	9.7 (8.0–11.7)	347	8.3 (6.3–10.9)	115
Other^a^	24.7 (22.2–27.4)	878	20.9 (19.3–22.5)	905
Marital status				
Married or cohabiting	70.7 (67.8–73.5)	2637	N/A	
Others^b^	29.3 (26.5–32.2)	897	N/A	
Living alone				
No	80.2 (77.6–82.5)	2947	74.4 (71.9–76.7)	2649
Yes	19.8 (17.5–22.4)	640	25.6 (23.3–28.1)	795
Underaged children in the household				
Yes	46.2 (43.2–49.3)	1121	34.5 (32.4–36.7)	2320
No	53.8 (50.7–56.8)	2320	65.5 (63.3–67.6)	1121
Language skills				
Beginner or less	32.0 (29.2–34.9)	1062	N/A	
Intermediate/excellent	68.0 (65.1–70.8)	2488	N/A	
Health-related factors				
Self-reported health				
Good or fairly good	71.4 (68.5–74.1)	2562	78.7 (77.0–80.3)	2582
Mediocre/fairly bad/bad	28.6 (25.9–31.5)	1013	21.3 (19.7–23.0)	841
Working capacity				
Full working capacity	82.0 (79.5–84.3)	2912	82.8 (81.2–84.4)	2719
Limited working capacity	18.0 (15.7–20.5)	653	17.2 (15.6–18.8)	703
BMI (kg/m²)				
Underweight/normal weight (<25)	45.1 (42.1–48.2)	1691	42.5 (40.0–45.1)	1288
Overweight (25–29.9)	37.1 (34.2–40.1)	1235	36.6 (34.4–38.8)	1317
Obese (⩾30)	17.8 (15.4–20.3)	599	20.9 (19.2–22.6)	783
Psychological distress^c^				
Psychological distress	19.9 (17.4–22.7)	638	10.2 (8.7–11.8)	330
No psychological distress	80.1 (77.3–82.6)	2831	89.8 (88.2–91.3)	3025
Quality of life				
Good (very good/good)	69.9 (67.0–72.7)	2541	78.7 (76.6–80.7)	2684
Other^d^	30.1 (27.3–33.0)	1003	21.3 (19.3–23.4)	744
COVID-19 related factors				
Contact with friends and relatives decreased^e^				
Yes	58.4 (55.3–61.4)	2149	59.3 (56.9–61.7)	2085
No	41.6 (38.6–44.7)	1418	40.7 (38.3–43.1)	1316
Loneliness increased^e^				
Yes	35.8 (32.9–38.8)	1277	27.3 (25.1–29.6)	890
No	64.2 (61.2–67.1)	2272	72.7 (70.4–74.9)	2499
Hope for the future decreased^e^				
Yes	38.1 (35.1–41.1)	1343	29.8 (27.6–32.1)	985
No	61.9 (58.9–64.9)	2185	70.2 (67.9–72.4)	2403
Worried about getting infected with COVID-19^f^				
Yes (very much/quite a lot)	28.3 (25.6–31.2)	1010	18.6 (17.1–20.2)	717
Moderately/no (moderately/a little/not at all)	71.7 (68.8–74.4)	2563	81.4 (79.8–82.9)	2685
Worried whether the employment of the respondent will continue during the epidemic^f^				
Yes (very much/quite a lot)	31.1 (28.3–34.1)	1045	7.8 (6.5–9.3)	237
Moderately/no (moderately/a little/not at all)	68.9 (65.9–71.7)	2474	92.2 (90.7–93.5)	3135
Worried about that someone close to the respondent will be infected with COVID-19^f^				
Yes (very much/quite a lot)	51.8 (48.7–54.9)	1864	40.8 (38.5–43.2)	1439
Moderately/no (moderately/a little/not at all)	48.2 (45.1–51.3)	1685	59.2 (56.8–61.5)	1963
Sleeping difficulties/nightmares increased^e^				
Yes	17.6 (15.3–20.1)	587	9.5 (8.0–11.2)	324
No	82.4 (79.9–84.7)	2956	90.5 (88.8–92.0)	3066
Current smoking				
Yes	20.0 (17.7–22.5)	649	17.6 (15.9–19.5)	620
No	80.0 (77.5–82.3)	2938	82.4 (80.5–84.1)	2824

*Note*: BMI: body mass index.

a ‘Other’ category includes retired on an old age pension, receiving a disability pension or rehabilitation benefit, on part-time retirement, unemployed or laid off, on family leave, or stay-at-home mother/father, and other.

b ‘Other’ category includes separated or divorced, widowed, and single.

c Mental Health Inventory-5 was utilised as the indicator for psychological distress. A score of 52 and under indicate psychological distress, while over 52 indicates no psychological distress. A detailed description of the variable is available in, for example, Holm et al. 2023, https://doi.org/10.1016/j.jpsychores.2022.111127.

d ‘Other’ category includes neither poor nor good, poor, and very poor.

e The question was as follows, with suboptions presented in the table: How has COVID-19 or its restrictive measures affected your everyday life (compared to the time before the epidemic)? Among persons of migrant origin, ‘Has COVID-19. . . on your everyday life?’.

f The question was as follows, with suboptions presented in the table: People may have concerns about coronavirus. Have you been worried about . . .?

### Analyses

For the first research question, frequencies and percentages were used to describe changes in smoking behaviour among current smokers. For the second research question, logistic regression with adjustments for age and sex was applied to test the differences between the two study groups in the proportion of those with decreased/increased smoking. For the third research question, multinomial binary logistic regression was utilised. First, univariate associations between each background variable and the outcome (increase/decrease vs. no change (ref.) in smoking behaviour) were assessed. The multivariable model was based on model selection by Akaike’s information criterion [22], where the variables with the best model fit were included. The results are reported as odds ratios (ORs) and 95% confidence intervals (95% CIs).

Inverse probability weights were used in all analyses, reducing non-response bias and taking into account unequal sampling probabilities. The weights were calculated separately for both samples. In addition, the stratification for both random samples were accounted for in the variance estimates.

## Results

Sex distribution was quite similar among persons of migrant origin and the general population, while the proportion of those in the younger age and the lower education group was higher compared with the general population (Table I).

### Changes in smoking behaviour

The age- and sex-adjusted results showed that among persons of migrant origin, 13.6% of current smokers decreased their smoking, 16.9% increased their smoking, while 69.5% had not changed their smoking behaviour (Table II). Corresponding rates for the general population were 9.2%, 11.7% and 79.1%, respectively. Considering persons of migrant origin, both increases and decreases in smoking were most prevalent among participants originating from Africa (excluding North Africa; hereafter referred to as Africa), Asia and Latin America.

**Table II.** Changes in smoking behaviour due to COVID-19 among current smokers, persons of migrant origin living in Finland by country/area and the general population, adjusted for age and sex.

	Decrease, %	*p* ^a^	No change, %	p^b^	Increase, %	*p* ^c^	Total, %	Number of observations
Russia or the former Soviet Union	14.8 (8.7–24.2)	0.111	70.5 (59.1–79.9)	0.100	14.7 (8.1–25.1)	0.471	100	205
Europe, North America, Oceania	7.4 (3.2–16.3)	0.649	77.6 (65.7–86.3)	0.765	15.0 (8.1–26.1)	0.435	100	205
Middle East and North Africa	15.9 (7.4–30.9)	0.179	66.6 (51.6–78.8)	0.064	17.6 (9.6–30.0)	0.228	100	114
Africa (excl. North Africa), Asia and Latin America	23.9 (12.4–41.2)	0.011	53.0 (36.7–68.6)	0.001	23.1 (12.1–39.6)	0.051	100	119
Persons of migrant origin, total	13.6 (9.6–19.0)	0.093	69.5 (63.0–75.2)	0.011	16.9 (12.7–22.2)	0.066	100	643
General population	9.2 (6.9–12.1)	ref.	79.1 (74.7–82.9)	ref.	11.7 (8.9–15.3)	ref.	100	610

a *P*-value of a *t*-statistic for logistic regression coefficient, testing the difference in decreased smoking between each group of persons of migrant origin and the general population.

b *P*-value of a *t*-statistic for logistic regression coefficient, testing the difference in unchanged smoking between each group of persons of migrant origin and the general population.

c *P*-value of a *t*-statistic for logistic regression coefficient, testing the difference in increased smoking between each group of persons of migrant origin and the general population.

The differences between decreases (*p* = 0.093) and increases (*p* = 0.066) in smoking between persons of migrant origin and the general population were statistically non-significant (Table II). A greater proportion of the respondents from Africa, Asia and Latin America decreased their smoking compared with the general population (p = 0.011). Supplemental Table S1 in Appendix B shows changes in smoking behaviour among current smokers by background variables.

### Correlates of changes in smoking behaviour

#### Persons of migrant origin

In univariate analyses, respondents from Africa, Asia and Latin America had higher odds of decreasing smoking compared with participants from Europe, North America and Oceania (Table III, Panel A, Model 1). Obesity was also associated with decreased smoking. In the multivariable model, originating from Russia or the former Soviet Union and Africa, Asia and Latin America, limited working capacity, and being worried about getting infected with COVID-19 were associated with decreased smoking, while those who were other than employed or students had lower odds of reporting a decrease in smoking (Table III, Panel A, Model 2).

**Table III.** Multinomial logistic regression between background factors and changed smoking behaviour (vs. no change) due to COVID-19 pandemic among current smokers, odds ratios (ORs) and 95% confidence intervals (95% CI) among persons of migrant origin living in Finland.

	PANEL A: Decrease				PANEL B: Increase			
	Model 1^a^		Model 2^b^		Model 1^a^		Model 2^b^	
	OR	95% CI	OR	95% CI	OR	95% CI	OR	95% CI
Sociodemographic factors								
Region of origin					N/A			
Europe, North America, Oceania	1.00		1.00		1.00			
Middle East and North Africa	1.92	0.54–6.75	2.20	0.70–6.94	1.34	0.52–3.48		
Russia or the former Soviet Union	2.23	0.74–6.70	**3.48**	1.19–10.18	1.01	0.38–2.66		
Africa (excl. North Africa), Asia and Latin America	**3.31**	1.02–10.7	**4.08**	1.35–12.37	1.96	0.67–5.72		
Age (years)								
21–34	0.17	0.17–1.90			**6.13**	2.08–18.1	**6.09**	1.40–26.48
35–44	0.14	0.14–1.88			1.87	0.57–6.14	0.69	0.16–3.02
45–54	0.19	0.19–2.15			2.46	0.73–8.37	2.25	0.49–10.38
55–66	1.00				1.00		1.00	
Sex								
Male	1.00				1.00		1.00	
Female	1.52	0.68–3.42			0.87	0.42–1.79	1.53	0.59–3.97
Education								
Comprehensive school or lower (Low)	1.00				1.00			
Upper secondary education (Middle)	0.97	0.35–2.67			2.12	0.75–6.00		
High education (High)	0.96	0.33–2.79			2.77	0.98–7.79		
Employment status								
Employed full-time or part-time	1.00		1.00		1.00		1.00	
Student	0.73	0.25–2.16	0.81	0.22–2.95	**3.06**	1.15–8.12	1.62	0.46–5.77
Other	0.42	0.13–1.33	**0.12**	0.03–0.49	1.70	0.75–3.88	1.88	0.70–5.05
Marital status								
Married or cohabiting	1.00				1.00		1.00	
Others	0.82	0.36–1.85			1.20	0.57–2.49	0.41	0.09–1.80
Living alone								
No	1.00		1.00		1.00		1.00	
Yes	0.99	0.40–2.43	0.52	0.20–1.38	1.35	0.63–2.92	2.40	0.52–11.12
Underaged children in the household								
No	1.00				1.00			
Yes	0.77	0.35–1.68			0.83	0.39–1.76		
Language skills								
Beginner or less	1.00				1.00		1.00	
Intermediate/excellent	0.88	0.39–2.01			0.57	0.28–1.18	0.51	0.24–1.08
Health-related factors								
Self-reported health								
Good or fairly good	1.00				1.00		1.00	
Mediocre/fairly bad/bad	1.10	0.48–2.57			1.72	0.83–3.56	2.02	0.80–5.10
Working capacity								
Full working capacity	1.00		1.00		1.00			
Limited working capacity	1.08	0.41–2.84	**3.29**	1.08–10.05	1.59	0.69–3.65		
BMI								
Underweight/normal weight	1.00		1.00		1.00		1.00	
Overweight	1.78	0.73–4.29	1.04	0.42–2.56	0.79	0.35–1.77	0.84	0.33–2.18
Obese	**2.81**	1.05–7.57	2.41	0.80–7.26	**0.34**	0.13–0.92	**0.34**	0.13–0.94
Psychological distress								
No psychological distress	1.00				1.00			
Psychological distress	1.25	0.50–3.16			1.62	0.73–3.60		
Quality of life								
Good	1.00				1.00			
Other	0.93	0.43–2.01			0.96	0.48–1.95		
COVID-19-related factors								
Contact with friends and relatives decreased								
No	1.00				1.00			
Yes	0.97	0.44–2.13			1.17	0.56–2.45		
Loneliness increased								
No	1.00				1.00			
Yes	1.51	0.67–3.42			1.63	0.79–3.37		
Hope for the future decreased								
No	1.00				1.00		1.00	
Yes	0.95	0.42–2.16			2.08	0.99–4.35	1.89	0.81–4.42
Worried about getting infected with coronavirus								
Moderately/No	1.00		1.00		1.00		1.00	
Yes	2.24	0.97–5.17	**3.51**	1.44–8.54	1.25	0.56–2.76	0.55	0.22–1.42
Worried whether the employment of the respondent will continue during the pandemic								
Moderately/No	1.00				1.00		1.00	
Yes	1.08	0.48–2.47			**2.38**	1.15–4.95	1.78	0.80–3.95
Worried about that someone close to the respondent will be infected with coronavirus								
Moderately/No	1.00		1.00		1.00		1.00	
Yes	1.11	0.50–2.46	0.49	0.20–1.21	**2.20**	1.13–4.27	2.31	0.98–5.45
Sleeping difficulties/nightmares increased								
No	1.00				1.00			
Yes	2.31	0.86–6.18			**2.83**	1.29–6.24		

*Note*: BMI: body mass index.

If the confidence interval of an odds ratio (OR) does not contain 1.00, the point estimate of the OR is presented in bold.

a Model 1: univarite association between background variable and change in smoking pattern.

b Model 2: multivariable association between background variable and change in smoking pattern; model selection by Akaike information criterion.

In the multivariable model, younger age was positively associated with increased smoking, while obesity was negatively associated with increased smoking (Table III, Panel B, Model 2).

#### General population

In the univariate model, those with limited working capacity had higher odds of decreased smoking (Table IV, Panel A, Model 1). In the multivariable model, limited working capacity and worries that someone close to them will be infected with COVID-19 were associated with higher odds of decreased smoking (Table IV, Panel A, Model 2).

**Table IV.** Multinomial logistic regression between background factors and changed smoking behaviour (vs. no change) due to COVID-19 pandemic among current smokers, odds ratios (ORs) and 95% confidence intervals (95% CI) among the general population.

	PANEL A: Decrease				PANEL B: Increase			
	Model 1^a^		Model 2^b^		Model 1^a^		Model 2^b^	
	OR	95% CI	OR	95% CI	OR	95% CI	OR	95% CI
Sociodemographic factors								
Age (years)								
21–34	0.58	0.23–1.46			1.73	0.73–4.09	**3.65**	1.33–9.97
35–44	1.06	0.53–2.13			1.83	0.89–3.77	**2.76**	1.03–7.37
45–54	0.75	0.35–1.59			1.76	0.85–3.67	**3.59**	1.39–9.30
55–66	1.00				1.00		1.00	
Sex								
Male	1.00				1.00		1.00	
Female	1.05	0.58–1.92			**2.37**	1.29–4.37	**2.28**	1.21–4.29
Education								
Low	1.00				1.00			
Middle	0.87	0.36–2.11			0.91	0.39–2.14		
High	0.82	0.31–2.13			1.60	0.66–3.88		
Employment status								
Employed full-time or part-time	1.00		1.00		1.00		1.00	
Student^c^	2.18	0.60–8.01	N/A		0.72	0.18–2.88	N/A	
Other	1.25	0.68–2.31	0.52	0.23–1.16	**2.02**	1.09–3.72	**3.38**	1.58–7.25
Living alone								
No	1.00				1.00			
Yes	1.50	0.81–2.75			0.97	0.51–1.85		
Underaged children in the household								
No	1.00				1.00			
Yes	1.20	0.63–2.29			1.36	0.73–2.51		
Health-related factors								
Self-reported health								
Good or fairly good	1.00				1.00			
Mediocre/fairly bad/bad	1.21	0.66–2.21			0.96	0.54–1.69		
Working capacity								
Full working capacity	1.00		1.00		1.00			
Limited working capacity	**2.43**	1.35–4.39	**2.69**	1.17–6.18	1.27	0.68–2.39		
BMI								
Underweight/normal weight	1.00				1.00		1.00	
Overweight	0.91	0.46–1.79			0.60	0.31–1.17	**0.46**	0.22–0.95
Obese	1.14	0.49–2.65			0.65	0.31–1.36	0.54	0.22–1.33
Psychological distress								
No psychological distress	1.00				1.00			
Psychological distress	1.00	0.45–2.19			1.93	0.94–3.95		
Quality of life								
Good	1.00				1.00			
Other	0.97	0.52–1.83			1.78	0.98–3.25		
COVID-19-related factors								
Contact with friends and relatives decreased								
No	1.00				1.00		1.00	
Yes	0.93	0.51–1.69			**2.99**	1.63–5.47	**2.53**	1.24–5.16
Loneliness increased								
No	1.00				1.00		1.00	
Yes	1.42	0.75–2.71			**5.29**	2.75–10.20	**5.57**	3.10–10.36
Hope for the future decreased								
No	1.00				1.00			
Yes	0.91	0.47–1.75			**1.93**	1.05–3.56		
Worried about getting infected with coronavirus								
Moderately/No	1.00				1.00			
Yes	1.82	0.98–3.37			**2.37**	1.29–4.35		
Worried whether the employment of the respondent will continue during the pandemic								
Moderately/No	1.00		1.00		1.00			
Yes	0.46	0.16–1.33	0.48	0.16–1.44	1.98	0.82–4.75		
Worried about that someone close to the respondent will be infected with coronavirus								
Moderately/No	1.00		1.00		1.00			
Yes	1.47	0.80–2.71	**2.19**	1.15–4.17	1.48	0.83–2.64		
Sleeping difficulties/nightmares increased								
No	1.00				1.00			
Yes	1.57	0.66–3.69			**4.79**	2.04–11.20		

*Note*: BMI: body mass index.

If the confidence interval of an odds ratio (OR) does not contain 1.00, the point estimate of the OR is presented in bold.

a Model 1: univariate association between background variable and change in smoking pattern.

b Model 2: multivariable association between background variable and change in smoking pattern; model selection by Akaike information criterion.

c Students in multivariable model reclassified into reference category to maximise the statistical power.

In the univariate model, COVID-19-related factors, such as increased loneliness, and female sex were associated with increased smoking (Table IV, Panel B, Model 1). In the multivariable model, younger age, female sex, being other than employed or a student, increased loneliness and decreased contacts with relatives and friends were associated with increased smoking (Table IV, Panel B, Model 2). Overweight was associated with decreased odds of increased smoking.

## Discussion

This study examined changes in current smoking behaviour due to COVID-19 among persons of migrant origin and the general population in Finland using population-based samples. Our study showed that the majority of smokers did not change their smoking behaviour due to COVID-19. Our investigation extends the current literature by indicating that a handful of the included sociodemographic-, health-, and COVID-19-related factors were associated with changes in smoking behaviour. These factors were predominantly different among persons of migrant origin and among the general population.

Our finding that the majority of smokers reported no change in their smoking behaviour corroborated the results of previous studies [14]. This proportion appeared to be higher among the general population than among persons of migrant origin, although the difference did not reach statistical significance. Thus, investing in the availability and acceptability of smoking cessation services is essential. Demand for remote cessation support increased during COVID-19 [23]. Telephone and website support should especially be developed in case of reduced possibilities for social contact in the health care system in the future [23]. Developing these services would be beneficial, particularly in regions comprising great geographical distances, such as Northern Finland, and for situations when face-to-face contact with others is not feasible.

Behavioural counselling and guaranteed financial incentives increase smoking cessation [24] and culturally adapted brief smoking cessation intervention can be effective [25]. Tailoring smoking cessation services to the needs of the target populations should be encouraged, since changes in smoking behaviour might differ across population groups. Inadequate evidence exists on whether culturally specific mass-media interventions are more effective in smoking-related behaviour change than campaigns targeted to the whole population [26]. Efficacy of culturally tailored interventions should be more thoroughly evaluated. When designing such interventions, appropriate and accessible channels for reaching the target population should be considered. The importance of selecting suitable strategies for promoting smoking cessation have also been reflected in previous research. For example, radio advertisements to call a quit line might increase such calls, especially among ethnic minorities, while evidence of the effect of culturally targeted smoking cessation booklets is more uncertain and limited to the USA among persons of African American origin [26]. Thus, the effects of smoking cessation interventions among different persons of migrant origin and in different focal countries warrants more research.

Our results showed that concerns related to the risk of COVID-19 infection were associated with a decrease in smoking both among persons of migrant origin and the general population. Among the former group, worries concerning getting infected with COVID-19 might encourage smokers to reduce their smoking, while for the latter group, concerns about a close person getting infected with COVID-19 might provide more relevant reasons for changes in smoking behaviour. Different living environments might play a role in interpreting COVID-19-related harms and possible behaviour change due to it. Persons of migrant origin are more likely to have a higher household density than Finnish-born persons [27]. Living environments might mirror the perceived risk of COVID-19 infection and other communicable diseases.

Limited working capacity was associated with decreased smoking among both persons of migrant origin and the general population. It might be the case that persons with limited working capacity also had commonly reported risk factors for COVID-19 infection, such as being overweight. Perceived increased risk of infection could then have resulted in an observed behaviour change. This interpretation is supported by the finding that among both persons of migrant origin and the general population, respondents with a high body mass index (BMI) were less likely to increase their smoking than those who were underweight/normal weight. People who stop smoking might gain some weight, which might discourage some smokers from attempting to quit [28]. Using nicotine replacement therapy (NRT) might reduce weight after smoking cessation, although the long-term effects are inconclusive [28]. Based on this notion, persons of migrant origin with a BMI of at least 30 might benefit from using NRT in their attempts to quit.

From the results from the current study, it is possible to distinguish the groups most at risk of increased smoking during crises such as COVID-19. Therefore, different enhanced preventive measures, as well as corrective actions, could be targeted at the at-risk groups. In our samples, younger age and female sex (only among the general population) were associated with increased smoking. Thus, these groups should be targeted in smoking prevention and smoking cessation services. The results of the current study were consistent with a previous study on changes in smoking behaviour during COVID-19 within the Italian population [13], as well as with a Finnish study conducted before the COVID-19 pandemic that showed a higher probability of smoking cessation among men and older age groups [29]. Our findings also corroborate prior results on the association between mental health aspects related to COVID-19 and increased smoking [11,13,18]; experiences of increased loneliness and reduced social contact due to COVID-19 were associated with increased smoking among the general population. These results imply that interventions that account for both psychological support, such as tackling loneliness, and supporting smoking cessation could have synergies. Most of the included factors showed non-statistically significant associations with changes in smoking behaviour due to COVID-19, most likely because of the limited sample size. This theme should be further explored both among persons of migrant origin and the general population. Longitudinal studies should also explore whether the impact of COVID-19 on smoking patterns was temporal or permanent.

### Limitations and strengths

The strengths of the study include the use of population-based samples both among persons of migrant origin and among the general population with highly comparable measures. No study, to the best of our knowledge, has examined changes in smoking behaviour among persons of migrant origin due to COVID-19, nor compared these changes and factors related to it with the general population. We were able to straightforwardly measure changes in smoking behaviour due to COVID-19 without the use of a proxy measure (for example, smoking behaviour change during COVID-19), thus reducing the effect of information bias. Sample weights were utilised to reduce response bias and improve the generalizability of the findings to the target populations.

Some limitations also need to be considered. Direct comparison between persons of migrant origin and the general population was not feasible in all of the analyses. However, the data protocols and included measures were highly similar, providing comparable information on changes in smoking behaviour. Smoking status and changes in smoking behaviour were self-reported and no biochemical verification was available. However, we were interested more in the changes in smoking behaviour than on the rate of smoking. Nonetheless, underreporting and the effect of non-participation cannot be ruled out, yet weighting will have corrected some of these effects. Changes in smoking behaviour were measured once during the second wave of the epidemic in Finland, yet these changes might have occurred several times during COVID-19. On the other hand, a Finnish study implied that there were few differences in changes in smoking behaviour between different time periods during the COVID-19 [15]. Even though the samples of the studies were large in comparison with previously published studies examining the impact of COVID-19 on smoking behaviour, particularly among persons of migrant origin, the sample size restricted some of the analyses. The impact of the sample size was particularly evident in the analysis by region of origin. The limited number of observations resulted in widened confidence intervals, reducing statistical power. With higher numbers of observations, some of the observed associations could have been stronger, such as the decreases/increases in smoking behaviour between persons of migrant origin and the general population.

## Conclusions

Most of the smokers among persons of migrant origin and the general population did not change their smoking behaviour due to COVID-19. The study demonstrated factors that are associated with health-enhancing and health-deterring behaviours. Culturally tailored smoking cessation services should be provided for population groups, where age, sex, and anxieties related to the crisis should be considered.

## Supplemental Material

sj-docx-1-sjp-10.1177_14034948231199792 – Supplemental material for Changes in smoking due to COVID-19 pandemic among persons of migrant origin compared with the general population: a population-based study Supplemental material, sj-docx-1-sjp-10.1177_14034948231199792 for Changes in smoking due to COVID-19 pandemic among persons of migrant origin compared with the general population: a population-based study by Otto Ruokolainen, Eero Lilja, Hanna Ollila, Anu E. Castaneda, Päivikki Koponen and Natalia Skogberg in Scandinavian Journal of Public Health

sj-docx-2-sjp-10.1177_14034948231199792 – Supplemental material for Changes in smoking due to COVID-19 pandemic among persons of migrant origin compared with the general population: a population-based study Supplemental material, sj-docx-2-sjp-10.1177_14034948231199792 for Changes in smoking due to COVID-19 pandemic among persons of migrant origin compared with the general population: a population-based study by Otto Ruokolainen, Eero Lilja, Hanna Ollila, Anu E. Castaneda, Päivikki Koponen and Natalia Skogberg in Scandinavian Journal of Public Health
