# Supplementary material for: Changes in smoking due to COVID-19 pandemic among persons of migrant origin compared with the general population: a population-based study
Source: Scand J Public Health. 2023 Sep 19;52(3):271–83. doi: 10.1177/14034948231199792 (PMC11067395; doi:10.1177/14034948231199792)
Supplement: sj-docx-1-sjp-10.1177_14034948231199792 – Supplemental material for Changes in smoking due to COVID-19 pandemic among persons of migrant origin compared with the general population: a population-based study [file sj-docx-1-sjp-10.1177_14034948231199792.docx]

### Supplemental Material, Appendix A Study instruments

Appendix 2: MigCOVID Survey questionnaire

**A-Posti Oy Posti Green**

**Impact of coronavirus epidemic on wellbeing among foreign born population**

(MigCOVID) Survey


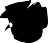


**INSTRUCTIONS FOR THE PARTICIPANT**

**X**

Please tick or write down your answers with a ballpoint pen in the space provided.

If you tick the wrong box, then please color in the whole box to cover the mistake and tick the box you intended.

Unless the instructions ask for more than one answer, please enter one option

**X** only per question that best describes your situation. Some questions have extra instructions for answering; please read these carefully before answering.

For more information, please contact:

[www.thl.fi/migcovid/info](http://www.thl.fi/migcovid/info)

toll-free number 0800 95335 (weekdays between 9-11 a.m. and 3-5 p.m.) or by e-mail [migco](mailto:migcovid@thl.fi)[vid@thl.fi](mailto:vid@thl.fi)

By answering the questionnaire, I give consent that my personal information will be handled as described in the data protection notification on handling personal data and that my answers can be linked with the findings of the FinMonik Survey, as well as register data on health and wellbeing. Answering is voluntary.

## Impact of the coronavirus on the daily life

1. People may have concerns about the coronavirus. Have you been worried about ...

| **not at all** | **a little** | **moderately** | **quite a lot** | **very much** |
| --- | --- | --- | --- | --- |
| Getting infected with coronavirus | | | | |
| Possibly infecting other people | | | | |
| Being discriminated against or avoided because you have coronavirus | | | | |
| Whether your employment will continue during the epidemic | | | | |
| The government's ability to deal with the coronavirus outbreak | | | | |
| The ability of the health care system to treat all coronavirus patients | | | | |
| That someone close to you will be infected with coronavirus | | | | |

1. **Has the corona epidemic or its restrictive measures affected your everyday life?**

*If there are things in the list that don't apply to your own life at all, select 'do not apply'.*

| **yes, yes, does not no effect decreased increased apply** |
| --- |
| Contact with friends and relatives |
| Loneliness |
| Disputes and conflicts within the family |
| Feeling of safety at home |
| Hope for the future |
| Daily physical activity levels (physical activity during commuting to work and leisure-time) |
| Smoking |
| Alcohol use |
| Sleeping difficulties, nightmares |
| Consumption of fruit, berries and vegetables (potato is not counted as a vegetable) |
| Snacks (consumption of sweets, chocolate, soft drinks, chips, etc.) |
| Doing remote work |
| Dealing with everyday chores online (e.g. online food purchases) |
| Online dealings with social welfare and health care services |

The following questions relate to your feelings of security and how you have been treated by others during the coronavirus epidemic.

1. *During the coronavirus epidemic:* **Have you been treated with less respect than others?**

No

Yes, at least once a week

Yes, less often than once a week

Has this happened:

more than before the coronavirus epidemic less than before the coronavirus epidemic no change

**5. *During the coronavirus epidemic:* Have you been threatened or harrassed?**

No

Yes, at least once a week

Yes, less often than once a week

Has this happened:

more than before the coronavirus epidemic less than before the coronavirus epidemic no change

**4. *During the coronavirus epidemic:* Have you been called names or verbally insulted?**

No

Yes, at least once a week

Yes, less often than once a week

Has this happened:

more than before the coronavirus epidemic less than before the coronavirus epidemic no change

1. Has the corona epidemic weakened your financial situation?

very much quite a lot

to some extent a little

not at all

## Impact of the coronavirus epidemic on health

1. Do you find that your current state of health is:

good

fairly good average fairly poor poor

1. Whether or not your are employed, rate your current work ability. Are you

completely able to work partially unable to work completely unable to work?

1. Do you have difficulty...

| **no difficulty** | **some difficulty** | **a lot of difficulty** | **cannot do it at all** |
| --- | --- | --- | --- |
| seeing ordinary newspaper print (with or without glasses) | | | |
| hearing what is said in a conversation between several people (with or without a hearing aid) | | | |
| walking about half a kilometre without resting | | | |

1. **How would you evaluate your memory? My memory works:**

very well well adequately poorly

very poorly

1. **How tall are you?** *Please round to nearest centimetre.*

cm

1. **How much do you weigh?** *Please round to nearest kilogramme.*

kg

1. Are you smoking currently (cigarettes, cigars or a pipe)?

yes, daily occasionally not at all

I have never smoked

1. The following symptoms may indicate being infected with the coronavirus. Have you experienced the following symptoms after March 1st 2020?

| **yes, during**  **the past 30 yes, earlier**  **days this year no** |
| --- |
| sore throat |
| a head cold |
| fever |
| cough |
| pain in the front part of your face |
| hoarseness |
| lost your voice |
| difficulties breathing |
| headache |
| muscle pain |
| pain when breathing in the middle of the chest and around the windpipe |
| a stabbing pain in the chest elsewhere than the windpipe |
| loss of your sense of smell or taste |
| bowel symptoms (e.g. diarrhoea, vomiting) |

1. **Do you think your symptoms were related to coronavirus disease?**

yes, I was diagnosed with a coronavirus infection I don't know for sure, but I think it's likely

no

1. Did you contact health care about your symptoms?

yes

no (skip to question 18)

1. What do you think of the treatment and instructions you received when you suspected you had coronavirus disease?

| **Completely Somewhat**  **agree agree Disagree I did not need** |
| --- |
| I was able to easily contact health care / the place of treatment |
| I received appointment time quickly |
| I easily got access to the test |
| I received enough information about the disease |
| I received clear instructions for treating the disease |

1. **If you did not seek care despite the symptoms, why did you not seek care?**

*You can choose one or more of the following alternatives.*

I felt that I did not need care

I did not know whom I should contact

My Finnish/Swedish/English skills are not sufficient for communicating in the healthcare setting I was worried what others would think if I were found to have a coronavirus infection

I have previously experienced discrimination in healthcare Too long waiting time for the coronavirus tests

Other reason, please specify:

1. In your opinion, have you received enough of the following services after March 1st 2020?

| **I would have I have used**  **needed it, but the service I have used the I have not did not receive but it was not service and it needed it the service adequate was adequate** |
| --- |
| doctor's appointment services |
| nurse's or public health nurse's appointment services |
| dentist services |
| Mental health care (by a psychologist, psychotherapist or a psychiatrist for example) |
| services for families with children (e.g. child welfare services, parenting and family counselling, home services) |

**Impact of the coronavirus epidemic on quality of life and mood**

When answering questions number ( 20- 21), please consider the past two weeks.

1. How would you rate your quality of life?

very poor poor

neither poor nor good good

very good

1. Below are listed some statements regarding emotions and thoughts. For each statement, please check the box that best describes your experiences in the past two weeks.

| **none of the time** | **rarely** | **some of the time** | **often** | **all of the time** |
| --- | --- | --- | --- | --- |
| I have felt hopeful about the future | | | | |
| I have felt useful | | | | |
| I have felt relaxed | | | | |
| I have dealt with problems well | | | | |
| I have thought clearly | | | | |
| I have felt closeness with other people | | | | |
| I have managed to make my own decisions on things | | | | |

1. **How much of the time during the past 4 weeks**

| **all of the time** | **most of the time** | **a good bit of the time** | **some of the time** | **a little bit of the time** | **none of the time** |
| --- | --- | --- | --- | --- | --- |
| Have you been a very nervous person? | | | | | |
| Have you felt so down in the dumps that nothing could cheer you up? | | | | | |
| Have you felt calm and peaceful? | | | | | |
| Have you felt downhearted and blue? | | | | | |
| Have you been a happy person? | | | | | |

**Receiving information during the coronavirus epidemic**

1. **From which source did you receive up-to-date information on the coronavirus epidemic?**

*You may choose more than one option per each row.*

| **in my mother I did not follow**  **in Finnish or tongue or this source of Swedish in English other language information** |
| --- |
| Finnish TV, radio, printed or digital newspaper |
| other country's (for example country of birth) or international TV channel, radio, printed or digital newspaper |
| Finnish authorities (for example municipality, InfoFinland.fi, THL, the Finnish Government) webpages or social media updates |
| other country's (for example country of birth) or international authority's webpages or social media updates |
| information or social media upfates by relatives, friends and aquaintances |
| information or social media updates by non- governmental organisations or associations, religious or other communities |
| other sources of information |

1. Which measures have you taken to avoid getting infected with the coronavirus and to prevent it from spreading?

| **Yes, I follow the I do not follow**  **instruction / the instruction / recommendation recommendation** |
| --- |
| I wash my hands more frequently |
| I use hand sanitizers more frequently |
| I take care of hygiene when coughing (e.g. coughing into a disposable tissue, not coughing into hands) |
| I stay at home if I have flu symptoms (e.g. cough, cold symptoms or sore throat) |
| I wear a single-use mask or cloth mask during my free time (when it is not possible to avoid close contact with other people) |
| I keep a 1 to 2 meter safe distance to other people outside of home |
| I do not shake hands with the people I meet |
| I do not take part in events with over 50 participants |
| I do not meet with people that are not part of my household |
| I do not travel outside of Finland |

1. **Have your received adequate information on how to avoid getting infected with the corona virus and how to prevent it from spreading?**

I have not received any information or the information I have received has been completely inadequate I have received information but I would have needed more

I have received adequate information

1. Have you downloaded the Koronavilkku contact tracing app to your mobile phone

yes

No, because the app is not available for my phone no, because I don't know what it is

no, because the app is not available in the languages I speak

no, for other reasons. Other reason?

## Background questions

1. Do you live alone?

yes no

1. **How many of your household members, including yourself, are the following ages.** *Please mark 0 for none.*

| **number** | | |
| --- | --- | --- |
| under 3 years old |  |  |
|  | | |
| 3-6 years old |  |  |
|  | | |
| 7-17 years old |  |  |
|  | | |
| 18-49 years old |  |  |
|  | | |
| 50-64 years old |  |  |
|  | | |
| 65-79 years old |  |  |
|  | | |
| 80 years old or older |  |  |

1. How many square meters is your home?

square meters

1. How many rooms are in your home?

number

1. What is the highest degree you have completed in Finland?

I have not attended education in Finland

lower than a comprehensive school degree (only a part of comprehensive school or similar)

comprehensive school degree matriculation examination

vocational qualification (e.g. a cook or a welder)

degree from a university of applied sciences (e.g. Bachelor of Social Services, Bachelor of Hospitality Management)

a bachelor's degree from a university

a master's degree from a university or a university of applied sciences

a licentiate or doctoral degree not sure

Which year did you complete

**this degree in Finland?**

1. **What is the highest degree you have completed abroad?**

I have not attended education abroad

lower than a comprehensive school degree (only a part of comprehensive school or similar)

comprehensive school degree matriculation examination

vocational qualification (e.g. a cook or a welder)

degree from a university of applied sciences (e.g. Bachelor of Social Services, Bachelor of Hospitality Management)

a bachelor's degree from a university

a master's degree from a university or a university of applied sciences

a licentiate or doctoral degree not sure

Which year did you complete

**this degree abroad?**

1. **Are you currently:**

married or in a registered relationship cohabiting

separated or divorced widowed

single

1. At the moment, are you principally:

*Please choose the option that best describes your situation*

employed full-time employed part-time

retired on an old age pension

receiving a disability pension or rehabilitation benefit on part-time retirement

unemployed or laid off

on family leave, or a stay-at-home mother/father a student

other

1. Which of the following statements describe your working conditions:

*You can choose one or more of the following alternatives.*

I am not working or in training

I work in health care, where I am in contact with clients I am able to keep 1-2m distance to others if I want

I can work remotely (from home)

I am able to take care of hand hygiene I have to come to work even if I am sick

1. Have you been laid-off or unemployed after March 1st 2020?

no

yes, lay-off/unemployment started before March 1st 2020

How long has it lasted in months? months

yes, lay-off started after March 1st 2020

How long has it lasted in months? months

yes, unemployment started after March 1st 2020

How long has it lasted in months? months

1. How well do you speak Finnish or Swedish?

not at all

beginner level: I am able to cope with simple everyday situations intermediate level: I am able to actively participate in conversations

excellent level: I am able to use Finnish diversely in different situations (e.g. I am able to manage issues with the authorities in Finnish)


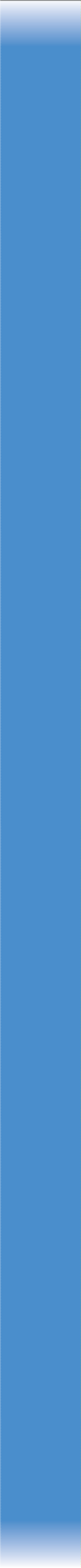


Separate the cover and survey from each other at the broken line


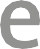

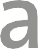


**A-Posti Oy Posti Green**


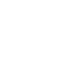

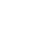


**thl.fi/finterveysseuranta**

User id:

Password:

FinHealth 2017 –study

**NATIONAL HEALTH SURVEY**

*Follow-up study 2020*

Dear

We recently sent you a letter regarding the FinHealth 2017- follow-up survey. According to our records, we have not yet received your response. You can respond to the survey online at **thl.fi/finterveysseuranta** with the user id and password found at the right top of this letter. After logging in, you can choose the language you prefer and begin responding to the survey. Alternatively you have the opportunity to respond to the attached paper questionnaire and post it in the included prepaid envelope.

We dearly hope that you take part in this survey. Every person selected for this survey is unique and cannot be replaced by another person.

**By responding, you can make a difference!**

Thank you for your cooperation! Kind Regards,

**More information on the study:**

- **online at**

**thl.fi/finterveysseuranta**

- **by e-mail at**

[**finterveysseurant**](mailto:finterveysseuranta@thl.fi)[**a@thl.fi**](mailto:a@thl.fi)

- **by phone (toll-free number): 080095332 on weekdays at 8-16**
- **for a privacy notice containing information about the processing of personal data in the survey, see thl.fi/finterveysseuranta**

Päivikki Koponen Research Manager

Source of address information: Population Information System, Digital and Population Data Services Agency, P.O. Box 123, FI-00531 Helsinki

**INSTRUCTIONS TO RESPONDENTS**

You should only choose one best alternative for each question unless it is specifically stated that you may choose more than one.

You may also fill in this questionnaire online at: [**http://www.thl.fi/finterveysseuranta**.](http://www.thl.fi/finterveysseuranta)

#### The online questionnaire requires your study number and password which can be found at the right top corner of the invitation letter.


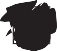


**X**

Tick the most suitable alternative or write the information required in the space given with a ballpoint pen. If possible, do not use a pencil.

**X**

In case you accidentally make markings into an unwanted box, please color in the entire box.

**X**

You should only choose one best alternative for each question unless it is specifically stated that you may choose more than one.

**X**

Remember to answer all questions. Enter negative answers by choosing the ‘no’ alternative or by writing ‘0’ (zero) in the space given.

By responding to this survey you consent to your personal data being processed in accordance with the privacy statement and to having your survey responses linked with health and welfare register data.

Participation in the study is voluntary.

**HEALTH STATUS**

##### Is your present state of health

###### good

rather good moderate rather poor poor

1. **Do you have any longstanding illness or health problem?**

no yes

1. **Are you limited because of a health problem in activities people usually do? Would you say you are**

severely limited

limited but not severely

not limited at all *(proceed to question 5)*

##### Have you been limited for at least the past 6 months?

###### yes no

1. **Have you during the past month (30 days) had the following symptoms or problems?**

| **less frequently**  **daily than daily not at all** |
| --- |
| Joint pain |
| Headache |
| Back pain |
| Urinary incontinence |
| Nausea |
| Dizziness |
| Toothache |

1. **When was the last time you used the following medication?**

*Please answer on every line by choosing the correct alternative.*

| **during the past 1-4 weeks 1-12 months over a year**  **week ago ago ago never** |
| --- |
| Painkillers for headache |
| Painkillers for joint or muscle ache |
| Painkillers for another ache |
| Sleeping pills |
| Tranquillizers |
| Antidepressants |
| Asthma medication |
| Allergy or hay fever medication |
| Diabetes medication (insulin and/or tablets) |
| Medicine for high blood pressure |
| Medicine to lower your cholesterol level |
| Acetylsalicylic acid to prevent myocardial infarction or cerebral infarction (e.g.  Aspirin, Disperin, Primaspan) |
| Medication to thin the blood, anticoagulants (Marevan, Pradaxa, Xarelto or Eliquis) |
| Antibiotics |

1. **Is the condition of your teeth and the health of your mouth at present**

good

rather good moderate rather poor poor

**MENTAL HEALTH**

1. **Have you during the past 12 months had a period of at least two weeks when, for most of the time**

you have lost interest in most things, such as hobbies, work or other things that usually give you pleasure?

you have been low-spirited or depressed?

**yes**

**no**

1. **How much of the time during the past 4 weeks**

| **all of the most of the a good bit of some of the a little bit of none of the time time the time time the time time** |
| --- |
| Have you been a very nervous person? |
| Have you felt so down in the dumps that nothing could cheer you up? |
| Have you felt calm and peaceful? |
| Have you felt downhearted and blue? |
| Have you been a happy person? |

1. **Below are some statements about feelings and thoughts. Please tick the box that best describes your experience of each over the last 2 weeks.**

| **none of the some of the all of time rarely time often the time** |
| --- |
| I’ve been feeling optimistic about the future |
| I’ve been feeling useful |
| I’ve been feeling relaxed |
| I’ve been feeling interested in other people |
| I’ve had energy to spare |
| I’ve been dealing with problems well |
| I’ve been thinking clearly |
| I’ve been feeling good about myself |
| I’ve been feeling close to other people |
| I’ve been feeling confident |
| I’ve been able to make up my own mind about things |
| I’ve been feeling loved |
| I’ve been interested in new things |
| I’ve been feeling cheerful |

Warwick-Edinburgh Mental Well-Being Scale (WEMWBS) © NHS Health Scotland, University of Warwick and University of Edinburgh, 2006, all rights reserved.

# FUNCTIONING

##### How do you manage the following activities nowadays?

| **without difficulties** | **with minor difficulties** | **with major difficulties** | **not at all** |
| --- | --- | --- | --- |
| to walk about half a kilometre without resting | | | |
| to run a short distance (about 100 metres) | | | |
| to climb one flight of stairs without resting | | | |
| to read an ordinary newspaper print (with or without glasses) | | | |
| to hear what is said in a conversation between several people (with or without a hearing aid) | | | |

1. **How would you estimate your present memory, learning capabilities, and ability to concentrate:**

| **very well** | **well** | **adequately** | **poorly** | **very poorly** |
| --- | --- | --- | --- | --- |
| How well does your memory work? | | | | |
| Are you able to acquire new information and learn? | | | | |
| Are you generally able to concentrate on matters? | | | | |

1. **Regardless of whether you are employed or not, please estimate your current work capacity. Are you:**

###### completely fit for work partially unable to work completely unable to work?

1. **Let’s assume that your work ability would receive a score of 10 points at its best. What point score would you give your current work ability?**

*Please choose the option that best applies to your working capacity.*

| **(completely (work**  **unable to work) ability at its best)** | | | | | | | | | | |
| --- | --- | --- | --- | --- | --- | --- | --- | --- | --- | --- |
| 0 | 1 | 2 | 3 | 4 | 5 | 6 | 7 | 8 | 9 | 10 |
|  | | | | | | | | | | |

1. **Over the past 12 months, how many whole days have you been absent from work or unable to do your chores due to illness?** *(If none, please enter the number "0".)*

###### days

1. **In terms of your health, do you feel that you will be able to work in your current profession until retirement age?** *If you are not employed at present, please answer as for your most recent job.*

###### I am already retired no

probably no probably yes yes

1. **What is your assessment of your ability to use the internet - online services (on computer or mobile devices)?**

I do not use them

novice/beginner (I use them with assistance) I use the basic services independently

I use many online applications effortlessly expert (I can teach others)

**WELL-BEING**

1. **Do you ever feel lonely?**

never

very rarely sometimes fairly often all the time

1. **How satisfied are you with your economic situation?**

very satisfied satisfied

somewhat satisfied unsatisfied

very unsatisfied

1. **How satisfied are you with your accomplishments in life?**

very satisfied satisfied

somewhat satisfied unsatisfied

very unsatisfied

1. **How satisfied are you with your family life?**

very satisfied satisfied

somewhat satisfied unsatisfied

very unsatisfied

I do not have a family

1. **In the future, do you wish to have (more) children?**

no

yes, but it is not yet/no longer an issue for us, or is not possible yes, I am/we are hoping or trying to conceive

I am, or my spouse or partner is currently pregnant I can't say

**LIFESTYLE**

1. **How much do you exercise and stress yourself physically in your leisure time?**

In my leisure time I read, watch TV and do other activities in which I do not move much and which do not strain me physically

In my leisure time I walk, cycle and move in other ways several hours a week. This includes walking, fishing and hunting, and light home gardening.

In my leisure time I exercise several hours a week. This includes running, jogging, cross country skiing, fitness training, swimming, ball games, and strenuous garden work.

In my leisure time I practice regularly strenuous sport several times per week. This includes competitive sports such as running, orienteering, cross country skiing, swimming and ball games.

1. **Do you think you sleep enough?**

yes, nearly always yes, often

rarely or hardly ever I can’t say

1. **Over the past month (30 days), how often have you...**

| **often sometimes not at all** |
| --- |
| felt excessively tired or sleepy during the daytime? |
| had nightmares |
| had trouble sleeping |

1. **How often have you eaten vegetables and root vegetables (not potatoes) during the past seven (7) days as such, grated or in fresh salads?**

not at all

on 1 to 2 days

on 3 to 5 days

on 6 to 7 days several times a day

1. **How often have you eaten fruits or berries during the past seven (7) days?**

not at all

on 1 to 2 days

on 3 to 5 days

on 6 to 7 days several times a day

1. **Do you smoke nowadays (cigarettes, cigars, pipefuls)?**

I have never smoked yes, daily

yes, occasionally

not at all, I quit smoking (month) / (year) *For example, if you quit smoking in July 2019, fill in 07 (month) 2019 (year). If you can´t recall the month, fill in only the year.*

##### Do you use snuff?

###### I have never used snuff yes, daily

yes, occasionally

not at all, I quit using snuff (month) / (year) *For example, if you quit using snuff in July 2019, fill in 07 (month) 2019 (year). If you can´t recall the month, fill in only the year.*

##### Do you currently use electronic cigarettes with nicotine?

###### I have never used them yes, daily

yes, occasionally

not at all, I quit using them (month) / (year)

*For example, if you quit using electronic cigarettes in July 2019, fill in 07 (month) 2019 (year). If you can´t recall the month, fill in only the year.*

1.
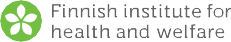
**How often do you have a drink containing alcohol?**

Never *(proceed to question 34)*

Monthly or less

2 to 4 times a month

2 to 3 times a week

4 or more times a week

1. **How many drinks containing alcohol do you have on a typical day when you are drinking?**


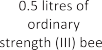

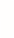

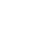

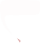

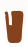

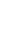

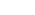

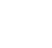

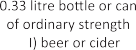

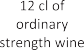

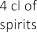

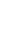

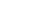

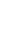

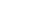


*Please see the figure for an example of alcohol units.*

1-2 units

3-4 units

5-6 units

7-9 units

10 units or more

1. **How often do you have six or more drinks on one occasion?**

never

less than monthly monthly

weekly

daily or almost daily

1. **Have any of the following people recommended you for health reasons in the past 12 months to.**

*(You may choose several alternatives on each row.)*

|  |  | **a public health** |  |  |
| --- | --- | --- | --- | --- |
|  |  | **nurse, or** |  |  |
| **no one** | **doctor or dentist** | **some other health care professional** | **family member** | **someone else** |
| exercise more | | | | |
| change your eating habits | | | | |
| lose weight | | | | |
| reduce consumption of alcohol | | | | |
| quit smoking | | | | |

**WEIGHT AND HEIGHT**

1. **How much do you weigh at present?**

kg (an estimate suffices)

1. **How tall are you?**

cm (an estimate suffices)

**SOCIAL WELFARE AND HEALTH CARE SERVICES**

1. **Over the past 12 months, how many times have you been to a doctor's appointment because of your own condition?** *Not including dentists' appointments (Mark 0 if not at all.)*

###### times

1. **Over the past 12 months, how many times have you been to a public health nurse's, nurse's or occupational health nurse’s appointment, or they have visited you at home because of your own condition?**

*(Mark 0 if not at all.)*

times

1. **Have you been in physiotherapy on a doctor’s referral during the past 12 months?**

no yes

1. **Which of the following do you primarily contact when in need of medical attention?**

*(Choose only one option)*

the health centre a private clinic

occupational health care student health care

a hospital outpatient clinic other

1. **Evaluate your experiences in your primary care facility in the past 12 months**

*(not including dental care).*

| **always** | **usually** | **sometimes** | **never** | **not applicable** |
| --- | --- | --- | --- | --- |
| I was able to get contact to the unit without difficulty | | | | |
| I had access to care without undue delay | | | | |
| I had access to examinations (laboratory tests, X ray imaging, ultrasound scans, etc.) without undue delay | | | | |
| Traveling to the care unit was difficult | | | | |
| High fees have made it more difficult for me to get care | | | | |

1. **When was the last time you visited a dentist?**

during the past 12 months 1 to 2 years ago

3 to 5 years ago over 5 years ago

I have never visited a dentist

1. **Did you get vaccinated against the flu for the season starting autumn 2019 and ending in the spring 2020?**

yes no

I can’t remember

**PROVIDING AND RECEIVING ASSISTANCE**

1. **Do you need and do you get help for your everyday activities due to your impaired functional capacity?**

I do not need help and do not get it I would need help but do not get it I get help, but not enough

I get enough help

I get more help than I need

1. **Do you yourself regularly help someone, who does not live in your household, who has limited functional capacity or is ill?**

no yes

1. **Do you regularly provide assistance to a person who is a member of your household and has limited functional capacity, or is ill, and could otherwise not cope at home?**

no

yes, my spouse or cohabitant yes, my child or grandchild

yes, my own or my spouse’s parents

yes, my own or my spouse’s grandparents some other person

1. **Estimate your possibilities to get help from people close to you when you need help or support.**

*You may choose more than one option per row.*

From whom do you get practical help when needed?

Who do you think really cares about you no matter what happened to you?

**husband, some close close**

**wife, other close fellow neigh- someone**

**partner relative friend worker bour else close no one**

**SOCIAL RELATIONSHIPS AND QUALITY OF LIFE**

1. **Do you currently have a close friend with whom you can talk confidentially about almost any issues concerning yourself?**

I don't have any close friends I have one close friend

I have two close friends

I have several close friends

1. **How much do you trust the following? On a scale from “I do not trust them at all” to “I trust them completely”, choose the option that best describes your opinion.**

| **I do not trust them at all**  1 | 2 | 3 | 4 | **I trust them completely**  5 |
| --- | --- | --- | --- | --- |
| public health services | | | | |
| public social services | | | | |
| courts of law | | | | |
| the police | | | | |
| decisionmaking in your municipality | | | | |
| people in general | | | | |

When answering questions number 50 to 52, think about your life **in the past two (2) weeks**.

##### How would you rate your quality of life?

###### very poor poor

neither poor nor good good

very good

1. **How satisfied are you with?**

| **very dissatisfied** | **dissatisfied** | **neither satisfied nor dissatisfied** | **satisfied** | **very satisfied** |
| --- | --- | --- | --- | --- |
| your health | | | | |
| your ability to perform your daily living activities | | | | |
| yourself | | | | |
| your personal relationships | | | | |
| the conditions of your living place | | | | |

1. **Do you have**

enough money to meet your needs

enough energy for everyday life

**completely**

**mostly**

**moderately**

**a little**

**not at all**

**CORONAVIRUS (COVID-19) EPIDEMIC**

1. **People may have concerns about the coronavirus. Have you been worried about ...**

| **not at all** | **a little** | **moderately** | **quite a lot** | **very much** |
| --- | --- | --- | --- | --- |
| ending up in quarantine | | | | |
| getting infected with coronavirus | | | | |
| possibly infecting other people | | | | |
| being discriminated against or avoided because you have coronavirus | | | | |
| whether your employment will continue during the epidemic | | | | |
| the economic impact of the coronavirus epidemic | | | | |
| shortages of food or other daily goods due to the coronavirus epidemic | | | | |
| the government's ability to deal with the coronavirus outbreak | | | | |
| the ability of the health care system to treat all coronavirus patients | | | | |
| that someone close to you will be infected with coronavirus | | | | |

1. **The following symptoms may indicate being infected with the coronavirus. Have you experienced the following symptoms after 1.3.2020?**

| **yes, during**  **the past 30 yes, earlier**  **days this year no** |
| --- |
| sore throat |
| head cold |
| fever |
| cough |
| pain in the front part of your face |
| hoarseness |
| lost your voice |
| difficulties breathing |
| headache |
| muscle pain |
| pain when breathing in the middle of the chest and around the windpipe |
| a stabbing pain in the chest elsewhere than the windpipe |
| loss of your sense of smell or taste |
| bowel symptoms (e.g. diarrhea, vomiting) |

1. **Have your received adequate information on how to avoid getting infected with the corona virus and how to prevent it from spreading?**

I have not received any information or the information I have received has been completely inadequate I have received information but I would have needed more

I have received adequate information

1. **Which measures have you taken to avoid getting infected with the coronavirus and to prevent it from spreading?**

| **Yes, I follow the I do not follow instruction / the instruction /**  **recommendation recommendation** |
| --- |
| I wash my hands more frequently |
| I use hand sanitizers more frequently |
| I take care of hygiene when coughing (e.g. coughing into a disposable tissue, not coughing into hands) |
| I stay at home if I have flu symptoms (e.g. cough, cold symptoms or sore throat) |
| I wear a single-use mask or cloth mask during my free time (when it is not possible to avoid close contact with other people) |
| I keep a 1 to 2 meter safe distance to other people outside of home |
| I do not shake hands with the people I meet |
| I do not take part in events with over 50 participants |
| I do not meet with people that are not part of my household |
| I do not travel outside of Finland |

1. **How has the corona epidemic or its restrictive measures affected your everyday life (compared to the time before the epidemic)?**

*If there are things in the list that don't apply to your own life at all, select 'do not apply'.*

| **yes, yes, does not no effect decreased increased apply** |
| --- |
| amount of time spent with family |
| contact with friends and relatives |
| loneliness |
| number of sex partners |
| disputes and conflicts within the family |
| fear of or experience of domestic violence or intimate partner violence |
| hope for the future |
| exercise during work commute |
| exercise in your free time |
| participation in the activities of any club, organization or association |
| smoking |
| alcohol use |
| sleeping difficulties, nightmares |
| number of meals and snacks eaten during the day |
| consumption of vegetables (cooked or uncooked, not including potatoes) |
| consumption of fruit and berries |
| snacks (consumption of sweets, chocolate, soft drinks, chips, etc.) |
| dealing with everyday chores online (e.g. online food purchases) |
| online dealings with social welfare and health care services |
| doing remote work |
| loss of job or temporary lay-off |
| helping someone close to you (e.g. by doing shopping for an elderly person belonging to a risk group) |

1. **Has the corona epidemic weakened your financial situation?**

very much quite a lot

to some extent a little

not at all

1. **Have you downloaded the Koronavilkku contact tracing app to your mobile phone**

yes

No, because the app is not available for my phone No, for other reasons?

**BACKGROUND QUESTIONS**

1. **What is your education?** *Mark your highest educational degree.*

###### elementary school, basic education lower secondary education vocational school/equivalent

upper secondary education/high school non-university lower education

Bachelor's Degree (university of applied science, college, or similar) Master's Degree (university degree, MA, or similar)

1. **How many members are there presently in your household (yourself included)?**

members

1. **How many of your household members including yourself are**

*(Please mark 0 for none.)*

under 3 years 3-6-years 7–17-years 18–64-years 65–79-years

80-years or older

1. **Which of the following alternatives best describes your current main activity:**

*(choose only one option)*

employed or self-employed (includes unpaid employment in a family-owned business, apprenticeship, and paid internship)

unemployed

student, further education, or unpaid internship retired

on family leave, or a stay-at-home mother/father other

1. **What kind of work duties (including study-related training) are you engaged in?**

I work in social and health care

I work in service tasks where I meet customers or in other work tasks where I cannot avoid close contact with colleagues

I work in another position where I can avoid close contact with customers and colleagues I do not work / I am not engaged in study-related training

**THANK YOU FOR YOUR RESPONSES!**
