# Supplementary material for: Changes in smoking due to COVID-19 pandemic among persons of migrant origin compared with the general population: a population-based study
Source: Scand J Public Health. 2023 Sep 19;52(3):271–83. doi: 10.1177/14034948231199792 (PMC11067395; doi:10.1177/14034948231199792)
Supplement: sj-docx-2-sjp-10.1177_14034948231199792 – Supplemental material for Changes in smoking due to COVID-19 pandemic among persons of migrant origin compared with the general population: a population-based study [file sj-docx-2-sjp-10.1177_14034948231199792.docx]

**Supplemental Material, Appendix B.**

**Supplemental Tables**

| **Supplemental Table SI. Changes in smoking behaviour due to COVID-19 among persons of migrant origin living in Finland and among the general population by background variables, %, unadjusted** | | | | | | | |
| --- | --- | --- | --- | --- | --- | --- | --- |
|  | Panel A: Persons of migrant origin | | | | Panel B: General population | | |
|  | Decrease | No change |  | Increase | Decrease | No change | Increase |
| **Socio-demographic factors** |  |  |  |  |  |  |  |
| **Region of origin** |  |  |  |  |  |  |  |
| Russia or the former Soviet Union | 15.2 (8.7–25.3) | 69.9 (58.1–79.5) |  | 14.9 (8.1–25.8) | N/A | N/A | N/A |
| Europe, North America, Oceania | 7.4 (3.2–16.5) | 77.8 (66.2–86.2) |  | 14.8 (8.0–25.6) | N/A | N/A | N/A |
| Middle East and North Africa | 13.4 (6.0–27.2) | 67.7 (53.4–79.3) |  | 18.9 (10.7–31.1) | N/A | N/A | N/A |
| Africa (excl. North Africa), Asia and Latin America | 21.0 (11.1–36.1) | 53.6 (37.6–69.0) |  | 25.3 (12.9–43.7) | N/A | N/A | N/A |
| Persons of migrant origin, total | 13.6 (9.6–19.0) | 69.5 (63.0-75.2) |  | 16.9 (12.7–22.2) | 9.2 (6.9–12.1) | 79.1 (74.7-82.9) | 11.7 (8.9–15.3) |
| **Age (years)** |  |  |  |  |  |  |  |
| 21-34 | 12.1 (6.7–21.0) | 59.6 (48.0–70.2) |  | 28.3 (19.0–40.0) | 6.8 (3.1–14.1) | 80.9 (69.8–88.6) | 12.3 (6.6–21.9) |
| 35-44 | 11.1 (5.3–21.9) | 78.1 (66.3–86.6) |  | 10.8 (5.6–19.9) | 11.7 (7.0–18.8) | 75.4 (67.1–82.2) | 12.9 (8.2–19.8) |
| 45-54 | 13.2 (7.0–23.5) | 73.1 (60.4–82.9) |  | 13.7 (6.8–25.6) | 8.6 (4.8–14.9) | 79.0 (71.0–85.2) | 12.5 (7.8–19.4) |
| 55-66 | 19.4 (8.1–39.7) | 74.5 (55.9–87.1) |  | 6.1 (2.4–14.3) | 11.1 (7.6–15.9) | 81.4 (75.7–86.0) | 7.5 (4.6–11.9) |
| **Sex** |  |  |  |  |  |  |  |
| Male | 11.5 (7.5–17.1) | 70.3 (62.3–77.3) |  | 18.2 (12.4–25.9) | 9.4 (6.4–13.6) | 83.4 (78.1–87.6) | 7.3 (4.6–11.3) |
| Female | 16.4 (9.2–27.7) | 67.4 (55.7–77.3) |  | 16.2 (9.8–25.4) | 9.8 (6.6–14.3) | 74.5 (67.9–80.1) | 15.7 (11.4–21.2) |
| **Education** |  |  |  |  |  |  |  |
| Low | 13.5 (6.4–26.3) | 77.2 (62.9–87.1) |  | 9.3 (4.1–20.0) | 10.9 (5.2–21.2) | 78.7 (67.4–86.8) | 10.4 (5.2–19.8) |
| Middle | 13.2 (7.8–21.3) | 68.9 (59.1–77.3) |  | 17.9 (11.3–27.4) | 9.6 (6.7–13.6) | 80.8 (75.4–85.2) | 9.6 (6.6–13.8) |
| High | 13.1 (7.2–22.6) | 64.8 (53.3–74.8) |  | 22.2 (14.3–32.8) | 9.1 (5.5–14.6) | 75.2 (67.3–81.7) | 15.8 (10.5–23.0) |
| **Employment status** |  |  |  |  |  |  |  |
| Employed full-time or part-time | 15.9 (10.7–22.9) | 70.0 (61.6–77.2) |  | 14.1 (9.1–21.4) | 8.6 (6.0–12.1) | 81.9 (77.2–85.8) | 9.5 (6.9–13.1) |
| Student | 12.1 (4.9–26.9) | 54.4 (34.6–72.8) |  | 33.5 (17.9–53.8) | 17.0 (5.6–41.5) | 75.9 (50.6–90.7) | 7.1 (2.0–22.4) |
| Other | 7.4 (2.7–18.6) | 70.7 (57.3–81.3) |  | 21.9 (12.7–35.0) | 10.5 (6.8–15.9) | 71.9 (63.6–79.0) | 17.5 (11.4–25.9) |
| **Marital status** |  |  |  |  |  |  |  |
| Married or cohabiting | 14.4 (9.5–21.2) | 68.8 (60.4–76.0) |  | 16.9 (11.5–24.1) | N/A | N/A | N/A |
| Others | 12.0 (6.5–21.2) | 68.4 (57.3–77.8) |  | 19.5 (12.0–30.2) | N/A | N/A | N/A |
| **Living alone** |  |  |  |  |  |  |  |
| No | 12.9 (8.6–18.8) | 71.1 (63.4–77.7) |  | 16.1 (11.0–22.9) | 8.5 (6.0–12.1) | 80.1 (75.3–84.3) | 11.3 (8.3–15.2) |
| Yes | 13.7 (7.0–25.0) | 65.2 (52.4–76.1) |  | 21.1 (12.6–33.2) | 12.3 (8.1–18.2) | 76.7 (68.7–83.1) | 11.0 (6.7–17.6) |
| **Under-aged children in the household** | |  |  |  |  |  |  |
| Yes | 10.5 (6.1–17.4) | 72.0 (61.5–80.6) |  | 17.5 (10.4–27.9) | 10.8 (6.6–17.0) | 76.0 (67.9–82.7) | 13.2 (8.4–20.1) |
| No | 14.7 (9.6–22.0) | 67.7 (59.2–75.2) |  | 17.5 (12.0–24.9) | 9.0 (6.5–12.4) | 80.7 (76.0–84.7) | 10.3 (7.5–14.0) |
| **Language skills** |  |  |  |  |  |  |  |
| Beginner or less | 13.3 (7.1–23.4) | 63.0 (51.2–73.4) |  | 23.7 (15.3–34.8) | N/A | N/A | N/A |
| Intermediate/excellent | 11.9 (7.9–17.5) | 73.0 (65.2–79.6) |  | 15.1 (9.9–22.4) | N/A | N/A | N/A |
| **Health-related factors** |  |  |  |  |  |  |  |
| **Self-reported health** |  |  |  |  |  |  |  |
| Good or fairly good | 12.8 (8.5–18.8) | 72.3 (64.5–79.0) |  | 14.9 (9.9–22.0) | 9.3 (6.6–13.0) | 79.1 (74.0–83.5) | 11.6 (8.3–15.8) |
| Mediocre/fairly bad/bad | 13.9 (7.4–24.7) | 62.9 (50.9–73.5) |  | 23.2 (14.8–34.4) | 11.0 (7.1–16.6) | 77.9 (71.3–83.3) | 11.1 (7.5–16.2) |
| **Working capacity** |  |  |  |  |  |  |  |
| Full working capacity | 12.8 (8.7–18.6) | 71.1 (63.8–77.4) |  | 16.1 (11.3–22.4) | 7.6 (5.2–10.9) | 81.7 (77.1–85.6) | 10.7 (7.8–14.4) |
| Limited working capacity | 13.8 (6.3–27.4) | 62.9 (47.6–76.0) |  | 23.4 (12.9–38.6) | 16.6 (11.4–23.5) | 70.1 (62.0–77.2) | 13.3 (8.3–20.6) |
| **BMI** |  |  |  |  |  |  |  |
| Underweight/normal weight | 8.2 (4.6–14.0) | 70.3 (60.5–78.6) |  | 21.5 (14.2–31.3) | 9.2 (5.8–14.4) | 76.5 (68.8–82.8) | 14.3 (9.6–20.7) |
| Overweight | 13.6 (7.7–23.1) | 68.6 (57.0–78.3) |  | 17.8 (10.3–28.8) | 8.4 (5.5–12.7) | 82.5 (76.6–87.2) | 9.0 (5.7–14.0) |
| Obese | 20.0 (10.2–35.3) | 71.4 (56.3–82.9) |  | 8.6 (3.9–17.9) | 10.4 (5.6–18.6) | 79.9 (71.1–86.5) | 9.7 (5.6–16.3) |
| **Psychological distress** |  |  |  |  |  |  |  |
| Psychological distress | 15.1 (7.3–28.8) | 62.9 (48.7–75.2) |  | 22.0 (12.6–35.6) | 9.7 (5.0–17.9) | 72.9 (59.8–83.0) | 17.4 (10.1–28.4) |
| No psychological distress | 12.5 (8.5–17.9) | 72.8 (65.6–78.9) |  | 14.8 (10.2–21.0) | 9.7 (7.2–13.0) | 80.5 (76.1–84.2) | 9.8 (7.2–13.3) |
| **Quality of life** |  |  |  |  |  |  |  |
| Good | 13.5 (8.5–20.8) | 68.6 (59.7–76.4) |  | 17.8 (11.7–26.3) | 9.7 (6.9–13.5) | 81.2 (76.3–85.2) | 9.1 (6.3–13.0) |
| Other | 12.7 (7.6–20.5) | 70.0 (60.2–78.3) |  | 17.3 (11.2–25.7) | 9.5 (5.9–14.8) | 75.3 (67.0–82.1) | 15.2 (10.2–21.9) |
| **COVID-19 related factors** |  |  |  |  |  |  |  |
| **Contact with friends and relatives decreased** | |  |  |  |  |  |  |
| Yes | 13.1 (8.4–19.9) | 68.1 (59.4–75.7) |  | 18.8 (12.9–26.6) | 9.3 (6.5–13.2) | 74.7 (68.3–80.2) | 16.0 (11.6–21.6) |
| No | 13.5 (7.8–22.2) | 70.0 (59.6–78.7) |  | 16.5 (9.9–26.3) | 9.9 (6.5–14.8) | 84.1 (78.7–88.3) | 6.0 (3.8–9.3) |
| **Loneliess increased** |  |  |  |  |  |  |  |
| Yes | 16.5 (9.1–27.9) | 61.4 (49.6–71.9) |  | 22.1 (14.3–32.5) | 11.9 (7.4–18.5) | 63.2 (52.4–72.9) | 24.9 (17.5–34.1) |
| No | 11.6 (7.6–17.2) | 73.6 (65.7–80.2) |  | 14.8 (9.6–22.2) | 8.6 (6.1–12.1) | 85.5 (81.1–88.9) | 5.9 (3.7–9.2) |
| **Hope for the future decreased** |  |  |  |  |  |  |  |
| Yes | 12.7 (6.9–22.1) | 63.3 (52.2–73.1) |  | 24.0 (15.9–34.5) | 9.1 (5.4–14.7) | 74.8 (65.9–82.0) | 16.1 (10.6–23.7) |
| No | 13.3 (8.7–19.9) | 73.5 (65.2–80.4) |  | 13.2 (8.2–20.6) | 9.9 (7.1–13.5) | 81.1 (76.4–85.0) | 9.1 (6.4–12.7) |
| **Worried about getting infected with coronavirus** | |  |  |  |  |  |  |
| Yes | 20.9 (11.6–34.8) | 59.0 (45.1–71.6) |  | 20.1 (11.4–33.0) | 14.4 (9.3–21.8) | 65.9 (56.7–74.1) | 19.6 (13.0–28.6) |
| Moderately/No | 10.5 (7.0–15.6) | 72.7 (65.4–79.0) |  | 16.7 (11.6–23.5) | 8.5 (6.1–11.8) | 82.2 (77.7–85.9) | 9.3 (6.7–12.8) |
| **Worried whether the employment of the respondent will continue during the epidemic** | | | | |  |  |  |
| Yes | 13.3 (7.3–22.8) | 61.5 (50.2–71.6) |  | 25.3 (16.7–36.3) | 5.0 (1.9–12.6) | 76.8 (60.6–87.7) | 18.3 (8.9–33.8) |
| Moderately/No | 12.4 (7.9–18.7) | 75.2 (67.3–81.7) |  | 12.4 (7.8–19.2) | 10.2 (7.6–13.4) | 79.7 (75.5–83.3) | 10.2 (7.7–13.3) |
| **Worried about that someone close to the respondent will be infected with coronavirus** | | | | |  |  |  |
| Yes | 13.4 (8.5–20.7) | 64.4 (54.9–72.9) |  | 22.2 (15.0–31.5) | 11.0 (7.6–15.8) | 75.5 (68.8–81.2) | 13.4 (9.3–19.0) |
| Moderately/No | 12.3 (7.1–20.3) | 76.3 (67.8–83.0) |  | 11.5 (7.6–17.0) | 7.8 (5.1–11.7) | 82.7 (77.5–86.9) | 9.5 (6.5–13.6) |
| **Sleeping difficulties/nightmares increased** |  |  |  |  |  |  |  |
| Yes | 22.4 (10.6–41.4) | 45.1 (29.7–61.5) |  | 32.5 (20.1–48.0) | 13.3 (6.5–25.3) | 56.2 (35.3–75.2) | 30.5 (16.7–48.8) |
| No | 11.1 (7.7–15.8) | 74.4 (67.4–80.2) |  | 14.5 (9.8–21.0) | 8.9 (6.6–12.0) | 82.7 (78.7–86.1) | 8.4 (6.1–11.5) |
